# Supplementary material for: Immune marker reductions in black and white Americans following sleeve gastrectomy in the short-term phase of surgical weight loss
Source: PLoS One. 2023 Jul 25;18(7):e0288868. doi: 10.1371/journal.pone.0288868 (PMC10368278; doi:10.1371/journal.pone.0288868)
Supplement: Attachment — Submitted filename: Immune marker reductions in black and white Americans following sleeve gastrectomy in the short term phase of surgical weight loss.pdf [file pone.0288868.s001.pdf]

PLOS ONE

Immune marker reductions in black and white Americans following sleeve gastrectomy in the short term phase of surgical weight loss

--Manuscript Draft--

**Manuscript Number:**	PONE-D-22-23672
**Article Type:**	Research Article
**Full Title:**	Immune marker reductions in black and white Americans following sleeve gastrectomy in the short term phase of surgical weight loss
**Short Title:**	Racial changes in inflammation after bariatric surgery
**Corresponding Author:**	Bernadette E. Grayson, PhD<br>University of Mississippi<br>Jackson, MS UNITED STATES
**Keywords:**	
**Abstract:**	Background Surgical weight loss procedures like vertical sleeve gastrectomy (SG) are sufficient in resolving obesity comorbidities and are touted to reduce the burden of pro-inflammatory cytokines and augment the release of anti-inflammatory cytokines. The goal was to determine if early changes to immunoglobulins and general markers of inflammation occur similarly after SG in Black Americans (BA) and White Americans (WA). Methods Personal information, anthropometric data, and plasma samples were collected from 58 participants (24 BA and 34 WA) before and 6 weeks after SG for the measurement of immunoglobulin A (IgA), IgG, IgM, C-reactive protein (CRP), and transforming growth factor (TGFβ). Results Collectively, IgG, TGFβ, and CRP were all significantly reduced at six weeks following SG. Both IgG and CRP were significantly elevated in BA in comparison to WA prior to weight loss. CRP levels in BA were reduced to a similar extent as WA, but IgG levels were more dramatically reduced in BA than WA despite the overall higher starting concentration. No change was observed in IgA and IgM. Conclusions These data suggest that SG improves markers of immune function in both BA and WA. More diverse markers of immune health should be studied in future work.
**Order of Authors:**	Charles L. Phillips, PhD
	Tran T. Le, MS
	Seth T. Lirette, PhD
	Bradley A. Welch, BS
	Sarah C. Glover, DO
	Adam Dungey, MBA, RN
	Kenneth D. Vick, MD
	Bernadette E. Grayson, PhD
**Additional Information:**	
**Question**	**Response**
**Financial Disclosure**<br><br>Enter a financial disclosure statement that describes the sources of funding for the work included in this submission. Review the submission guidelines for detailed requirements. View published research articles from *PLOS ONE* for specific examples.	MS-CEPR)-COBRE P20GM121334 BEG SL<br>1U54GM115428 BEG SL

This statement is required for submission and **will appear in the published article** if the submission is accepted. Please make sure it is accurate.<br><br>**Unfunded studies**<br>Enter: *The author(s) received no specific funding for this work.*<br><br>**Funded studies**<br>Enter a statement with the following details:<br>• Initials of the authors who received each award<br>• Grant numbers awarded to each author<br>• The full name of each funder<br>• URL of each funder website<br>• Did the sponsors or funders play any role in the study design, data collection and analysis, decision to publish, or preparation of the manuscript?<br>• **NO** - Include this sentence at the end of your statement: *The funders had no role in study design, data collection and analysis, decision to publish, or preparation of the manuscript.*<br>• **YES** - Specify the role(s) played.<br><br>* typeset	
**Competing Interests**<br><br>Use the instructions below to enter a competing interest statement for this submission. On behalf of all authors, disclose any competing interests that could be perceived to bias this work—acknowledging all financial support and any other relevant financial or non-financial competing interests.<br><br>This statement is required for submission and **will appear in the published article** if the submission is accepted. Please make sure it is accurate and that any funding sources listed in your Funding Information later in the submission form are also declared in your Financial Disclosure statement.<br><br>View published research articles from *PLOS ONE* for specific examples.	The authors have declared that no competing interests exist.

**NO authors have competing interests**<br><br>Enter: *The authors have declared that no competing interests exist.*<br><br>**Authors with competing interests**<br><br>Enter competing interest details beginning with this statement:<br><br>*I have read the journal's policy and the authors of this manuscript have the following competing interests: [insert competing interests here]*<br><br>* typeset	
**Ethics Statement**<br><br>Enter an ethics statement for this submission. This statement is required if the study involved:<br><br>• Human participants<br>• Human specimens or tissue<br>• Vertebrate animals or cephalopods<br>• Vertebrate embryos or tissues<br>• Field research<br><br>Write "N/A" if the submission does not require an ethics statement.<br><br>General guidance is provided below. Consult the submission guidelines for detailed instructions. **Make sure that all information entered here is included in the Methods section of the manuscript.**	. All procedures were performed in accordance per the 1964 Declaration of Helsinki ethical standards. Written informed consent was obtained from each participant before formally entering this IRB approved study, Predictors of Weight Loss (POWL), at the University of Mississippi Medical Center (UMMC), Jackson, MS (IRB# 2014-0047).

Powered by Editorial Manager® and ProduXion Manager® from Aries Systems Corporation

**Format for specific study types**<br><br>**Human Subject Research (involving human participants and/or tissue)**<br>• Give the name of the institutional review board or ethics committee that approved the study<br>• Include the approval number and/or a statement indicating approval of this research<br>• Indicate the form of consent obtained (written/oral) or the reason that consent was not obtained (e.g. the data were analyzed anonymously)<br><br>**Animal Research (involving vertebrate animals, embryos or tissues)**<br>• Provide the name of the Institutional Animal Care and Use Committee (IACUC) or other relevant ethics board that reviewed the study protocol, and indicate whether they approved this research or granted a formal waiver of ethical approval<br>• Include an approval number if one was obtained<br>• If the study involved *non-human primates*, add *additional details* about animal welfare and steps taken to ameliorate suffering<br>• If anesthesia, euthanasia, or any kind of animal sacrifice is part of the study, include briefly which substances and/or methods were applied<br><br>**Field Research**<br><br>Include the following details if this study involves the collection of plant, animal, or other materials from a natural setting:<br>• Field permit number<br>• Name of the institution or relevant body that granted permission	
**Data Availability**<br><br>Authors are required to make all data underlying the findings described fully available, without restriction, and from the time of publication. PLOS allows rare exceptions to address legal and ethical concerns. See the PLOS Data Policy and FAQ for detailed information.	Yes - all data are fully available without restriction

A Data Availability Statement describing where the data can be found is required at submission. Your answers to this question constitute the Data Availability Statement and **will be published in the article**, if accepted.<br><br>**Important:** Stating 'data available on request from the author' is not sufficient. If your data are only available upon request, select 'No' for the first question and explain your exceptional situation in the text box.<br><br>Do the authors confirm that all data underlying the findings described in their manuscript are fully available without restriction?	
**Describe where the data may be found in full sentences. If you are copying our sample text, replace any instances of XXX with the appropriate details.**<br><br>• If the data are **held or will be held in a public repository**, include URLs, accession numbers or DOIs. If this information will only be available after acceptance, indicate this by ticking the box below. For example: *All XXX files are available from the XXX database (accession number(s) XXX, XXX.)*.<br>• If the data are all contained **within the manuscript and/or Supporting Information files**, enter the following: *All relevant data are within the manuscript and its Supporting Information files.*<br>• If neither of these applies but you are able to provide **details of access elsewhere**, with or without limitations, please do so. For example:<br><br>*Data cannot be shared publicly because of [XXX]. Data are available from the XXX Institutional Data Access / Ethics Committee (contact via XXX) for researchers who meet the criteria for access to confidential data.*<br><br>*The data underlying the results presented in the study are available from (include the name of the third party*	All relevant data are within the manuscript and its Supporting Information files.

and contact information or URL).<br>• This text is appropriate if the data are owned by a third party and authors do not have permission to share the data.<br><br>* typeset	
Additional data availability information:	Tick here if the URLs/accession numbers/DOIs will be available only after acceptance of the manuscript for publication so that we can ensure their inclusion before publication.

Powered by Editorial Manager® and ProduXion Manager® from Aries Systems Corporation

Click here to access/download;Manuscript;Phillips POM upload.docx

Title: Immune marker reductions in black and white Americans following sleeve gastrectomy in the short term phase of surgical weight loss

AUTHORS: [1,2]Charles L. Phillips, BS; [3]Tran T. Le, MS [3]Seth T. Lirette, PhD; [1]Bradley A. Welch, BS, [4]Sarah C. Glover, DO; [5]Adam Dungey, MBA, RN [5]Kenneth D. Vick, MD; and [1]Bernadette E. Grayson PhD

AFFILIATIONS:

[1]Program in Pathology
[2]Department of Neurobiology and Anatomical Sciences
[3]Department of Data Science
[4]Department of Internal Medicine
[5]Department of Surgery

INSTITUTION: University of Mississippi Medical Center, Jackson, MS

CORRESPONDENCE

Bernadette E. Grayson, Ph.D.
Associate Professor
University of Mississippi Medical Center
Department of Neurobiology and Anatomical Sciences
2500 North State Street
Jackson, MS 39216
Email: bgrayson@umc.edu
Phone: 601-984-6809
Fax: 601-984-1655

Running Title: Racial changes in inflammation after bariatric surgery

ABSTRACT

Background. Surgical weight loss procedures like vertical sleeve gastrectomy (SG) are sufficient in resolving obesity comorbidities and are touted to reduce the burden of pro-inflammatory cytokines and augment the release of anti-inflammatory cytokines. The goal was to determine if early changes to immunoglobulins and general markers of inflammation occur similarly after SG in Black Americans (BA) and White Americans (WA). **Methods**. Personal information, anthropometric data, and plasma samples were collected from 58 participants (24 BA and 34 WA) before and 6 weeks after SG for the measurement of immunoglobulin A (IgA), IgG, IgM, C-reactive protein (CRP), and transforming growth factor (TGFβ).

Results. Collectively, IgG, TGFβ, and CRP were all significantly reduced at six weeks following SG. Both IgG and CRP were significantly elevated in BA in comparison to WA prior to weight loss. CRP levels in BA were reduced to a similar extent as WA, but IgG levels were more dramatically reduced in BA than WA despite the overall higher starting concentration. No change was observed in IgA and IgM.

Conclusions. These data suggest that SG improves markers of immune function in both BA and WA. More diverse markers of immune health should be studied in future work.

Keywords: immune markers, bariatric surgery, vertical sleeve gastrectomy, immunoglobulins, weight loss surgery

INTRODUCTION

Obesity and its associated comorbidities continue to present a strain on health care around the world. Obesity is a disease of excess adiposity accompanied by chronic low-grade inflammation. The expanding size and number of adipocytes stress the surrounding tissues causing active secretion of cytokines from the adipose and endothelial cells and infiltration of immune cells into the surrounding tissues. Over time, inflammatory markers are elevated enough to be reliably measurable in circulation. The elevated inflammation further drives both systemic and local immune cell activation and cytokine secretion resulting in diverse negative impacts on various organ systems including immuno-hematologic parameters.

Black Americans (BA) carry a larger burden of obesity-related diseases than White Americans (WA). BA women have the greatest prevalence of obesity in the United States [1]. Among BA women, 80% are overweight or obese in comparison to only 62.4% of WA women [2]. 34.6% of AA women are Class II and III Obese compared to 17.6% of WA women [2]. Further, among BA men and women, the age-adjusted prevalence of hypertension is 39.6% and 43.1%, respectively [3]. In WA men and women, the prevalence is 31.4% and 28.7%, respectively [3]. Racial disparities also exist in the incidence of T2DM; 18.7% of all BA >20 years of age have T2DM compared to 7.1% of WA [4]. As a whole, BA suffer from a higher rate of metabolic-related diseases in comparison to WA.

In the U.S., BA also carry a higher burden of inflammation than WA. In a study of racial and ethnic disparities in children, the risk of inflammation is higher in BA children in comparison to WA children [5]. Low parental education and elevated child BMI were

partial mediators of this higher association [5]. In a cross-sectional investigation of ethnicity and blood levels of inflammatory markers in non-smoking, church-goers, BA had higher CRP and IL-6 than WA [6]. Further, in a study comparing inflammatory markers and breast cancer risk factors, BA women had higher levels of inflammatory cytokines IL-6 and interferon γ [7]. In a study of the effect of sleep and loss of immunity, BA participants had higher levels of Il-6 and IL-10 than WA [8]. Taken together, significant evidence in a variety of different sectors of population suggests that BA have higher levels of inflammatory mediators than WA.

Surgical weight loss procedures are sufficient in the resolution of obesity comorbidities such as excess weight, diabetes, and hypertension [9]. However, recent studies suggest that BA have reduced improvements to body weight loss and percentage of total weight loss in comparison to WA [10]. Higher overall rates of complications and health care resource utilization in BA have also been reported within 30 days of surgery in comparison to controls [10]. Bariatric surgeries are touted to reduce the burden of pro-inflammatory cytokines and augment release of anti-inflammatory cytokines [11,12]. Immunoglobulins (Ig) are reduced by bariatric surgery [13] in addition to several inflammatory cytokines and markers levels [14,15]. Moreover, bariatric surgery alters immune cell function and proliferation [16-18]. Nevertheless, the impact of surgery on inflammation in BA surgical recipients has not been reported.

Given that the burden of obesity-related disease is substantial in BA, and inflammation is higher in BA independent of this, understanding the effect of surgical weight loss outcomes in BA is important. The focus of this study was to determine if bariatric surgery ameliorates obesity-related inflammation in BA to a similar extent as

seen in WA subjects. Vertical sleeve gastrectomy (SG) is the most common surgery currently performed in the U.S. Here we report some key differences in the early changes of inflammatory markers after SG in a patient population of Mississippi, a state with one of the highest burdens of obesity-related comorbidities. Using anthropometric data and plasma samples from patients before and six weeks following SG, we investigated specific inflammatory markers IgG, IgM, IgA, TGFβ, and CRP.

MATERIALS AND METHODS

Assurances. All procedures were performed in accordance per the 1964 Declaration of Helsinki ethical standards. Written informed consent was obtained from each participant before formally entering this IRB approved study, Predictors of Weight Loss (POWL), at the University of Mississippi Medical Center (UMMC), Jackson, MS (IRB# 2014-0047).

Study Design. This is a non-randomized prospective study of obese patients receiving elective SG surgery through the Weight Management Clinic, UMMC were consented. The inclusion criteria for this study comprised of men and women between the ages of 21-65 years, BMI ≥35 kg/m^2, and undergoing first-time bariatric procedures between June and December 2016. Exclusion criteria for participation in this study were as follows: 1) Individuals with major organ system failure like: cirrhosis, hepatic insufficiency, portal hypertension, severe renal insufficiency or on dialysis, severe arterial insufficiency, dementia, or the inability to give informed consent. 2) Individuals who are pregnant or lactating. 3) Individuals with prior surgical weight loss procedure.

REDCap electronic record. Study data were collected and managed using REDCap (Research Electronic Data Capture) tools [19] hosted at the University of Mississippi Medical Center. REDCap is a secure, web-based application designed to support data

capture for research studies, providing 1) an intuitive interface for validated data entry; 2) audit trails for tracking data manipulation and export procedures; 3) automated export procedures for seamless data downloads to common statistical packages; and 4) procedures for importing data from external sources.

Plasma Analytes**.** Blood was collected in EDTA coated tubes, processed for plasma and stored at -80 ºC until further use. The following kits were used: C-reactive protein (#80955, CrystalChem, Elk Grove Village, IL), immunoglobulin A (#88-50600, Thermofisher, Waltham, MA), immunoglobulin G (#88-50550, Thermofisher, Waltham, MA), immunoglobulin M (#88-50620, Thermofisher, Waltham, MA), and tumor growth factor beta (#RAB0460, SigmaAldrich, St. Louis, MO). All assays were performed according to the manufacturers' specifications.

Statistical Analysis. Descriptive statistics were compiled where appropriate. For comparing post vs. pre-operative characteristics of participants, paired student's T tests and two-way analysis of variance with repeated measures for time were used followed by Tukey's post hoc test for variables of race and time with results are given as means ± SEM. Models for pre-op immune markers consisted of generalized linear models with gamma families to account for right skewness of the markers and identity link to facilitate ease of interpretation. In addition to the adjusters listed below, these models were adjusted for pre-op systolic and diastolic blood pressures. Weight change was modeled using ordinary least squares on the difference in weight post-op vs. pre-op. Similar models were used to model immune marker change, additionally adjusting for BMI change, systolic change, and diastolic change. All models were adjusted for age, sex, race, hypertension, hyperlipidemia, sleep apnea, and diabetes. Multivariable

fractional polynomials were used as a first step in modeling to account for potential nonlinearities. All linearity assumptions were found to be valid. Results were considered statistically significant when $p < 0.05$. All statistical analyses were performed with GraphPad Prism v8.1.2 (GraphPad Software, San Diego, California) and Stata v16.1 (StataCorp, College Station, Texas).

RESULTS

Baseline Characteristics of Participants. The total number of participants for the current study was 58 individuals of which 24 self-identified as BA and 34 self-identified as WA. Of the BA participants, all 24 were female, comprising 41.3% of the total; we were unable to enroll any BA males. Of the 34 WA participants, 28 or 48.2% were females and 6 participants or 10.3% of the total were WA males. There was no difference in the average age of female BA and WA participants (average age ± SEM, 45.5±2.0 years and 46.9±2.0 years respectively) or WA males (average age of 43.8±2.7 years)**.** BA participants had significantly higher levels of hypertension in comparison to WA patients, $p<0.001$, (**TABLE 1**)**.** No difference was measured in the instances of hyperlipidemia, diabetes, or obstructive sleep apnea between BA and WA **(TABLE 1).**

Pre-operative Characteristics of BA and WA Participants. There were no differences in pre-operative body weight or BMI of BA vs. WA participants enrolled in the study (**TABLE 2**). Further, there was no difference in waist circumference of BA and WA participants (**TABLE 2**); however, hip circumference was significantly higher pre-operatively in BA, $p<0.05$ (**TABLE 2**). Blood pressure and pulse did not differ significantly between BA and WA (**TABLE 2**). Liver enzymes, ALT and AST, were significantly higher in WA participants compared to BA, although there was no

difference observed in bilirubin, $p<0.001$ (**TABLE 2**). Creatinine and glucose were not different between BA and WA (**TABLE 2**). There was no difference in WBC count, though hematocrit percentage was significantly higher in WA, $p<0.01$, and platelets were significantly higher in BA, $p<0.05$ (**TABLE 2**).

Pre-operative Inflammatory markers in BA and WA Participants. Age did not influence inflammatory markers CRP, IgA, IgG, IgM, or TGFβ at the pre-operative time point (**TABLE 3**). BA participants had on average 1377.36 mg/dL higher IgG levels than WA participants pre-operatively, $p=0.001$ (**TABLE 3**). There was no influence of hypertension or sleep apnea status on the inflammatory markers tested pre-operatively (**TABLE 3**). However, the presence of diabetes was associated with a 17.33 mg/dL reduction on average of IgA in comparison to non-diabetics, $p<0.05$, and hyperlipidemia was associated with 1.20 mg/dL lower CRP, $p=0.001$ (**TABLE 3**).

Pre-operatively, BMI did not influence the circulating levels of immune markers measured in this cohort (**TABLE 3**). Additionally, pre-operative systolic blood pressure was not an influence on circulating levels of immune markers (**TABLE 3**). However, higher pre-operative diastolic blood pressure was associated with a significant, albeit subtle, reduction in circulating pre-operative CRP levels by 0.04 mg/L, $p<0.05$ (**TABLE 3**).

Comparison of BA and WA six weeks after SG.. As anticipated, 6 weeks following SG, both BA and WA lost a significant amount of body weight (p<0.0001), with no difference in weight change identified by race (**TABLE 2**). When percent excess body weight (%EWL) is calculated, based on ideal weight, WA participants (Mean ±SEM) (20.55 ± 1.327) had a greater amount of excess bodyweight loss in comparison to BA

participants (24.15 ± 0.86), $p<0.05$. Percent weight loss (%WL) was not significant (BA 11.24 ± 0.56 vs. WA 12.44 ± 0.34), nor was the change in BMI (BA 5.32 ± 0.27 vs. WA 5.547 ± 0.20) or percent excess BMI loss (%EBMIL) (BA 25.2 ± 2.07 vs. WA 29.53 ± 1.15).

Further, BMI ($p<0.0001$), waist circumference ($p<0.0001$), and hip circumference ($p<0.0001$) were all reduced at 6 weeks post SG with no differences identified by race (**TABLE 2**). Pulse rate ($p<0.0001$) and systolic blood pressure ($p<0.0001$) significantly reduced following SG with no differences specified by race (**TABLE 2**). Diastolic blood pressure did not change six weeks after SG as a function of time (**TABLE 2**). However, a significant racial difference in diastolic blood pressure at 6 weeks post-surgery was measured with BA having less of a reduction than WA, $p<0.05$ (**TABLE 2**).

Overall, weight and BMI changes did not correlate with race or the presence of the comorbidities we reported, such as hypertension, hyperlipidemia, sleep apnea, or diabetes mellitus (**TABLE 3**). On the other hand, greater reduction in both weight and BMI were correlated with age, such that younger participants lost weight at an average of 0.10 kg more per year younger and 0.04 points of BMI per year younger than older participants, $p<0.05$ (**TABLE 3**).

<u>Effect of SG on circulating immune markers.</u> Plasma IgA and IgM were not altered because of SG (**FIGURE 1**), and there was no impact of race on immunoglobulin A and M levels (**FIGURE 1**). Alternately, there was a significant reduction in plasma IgG after SG, $p<0.001$ (**FIGURE 1**). Plasma IgG varied significantly by race with BA having higher levels of IgG than WA, $p<0.001$ (**FIGURE 1**). Furthermore, BA had greater reductions in IgG, with an average reduction of 1108 mg/dL as a result of SG in comparison to WA

(**TABLE 5**). Age did not impact the change of any of the cytokines during the first six weeks post-operatively, except for TGFβ, such that for every year older, there was a 0.35 mg/dL increase in TGFβ, $p<0.05$ (**TABLE 5**).

The presence of pre-operative hypertension or sleep apnea status did not contribute to changes in any of the immune markers measured (**TABLE 5**). However, in participants who had diabetes mellitus pre-operatively, SG promoted a greater change, on average, of 23.81 mg/dL in IgM levels than those who did not have diabetes, $p<0.05$ (**TABLE 5**). Change over the 6 weeks following SG in BMI, systolic blood pressure, and diastolic blood pressure did not correlate significantly with any of the shifts in the markers of inflammation tested (**TABLE 5**).

DISCUSSION

Obesity is a condition of excess adiposity that results in chronic low-grade inflammation. Surgical weight loss procedures produce robust improvements in metabolic indices and increase the quality and longevity of life for the obese individual that is burdened with an array of metabolic comorbidities [20]. In the current study, we asked whether inflammatory markers were altered in BA SG recipients differentially than in WA participants and whether these changes accounted for any variation in change in BMI in the six week time frame following bariatric surgery.

Pre-operatively, BA and WA participants were not significantly different overall; however, there were a few notable differences between the groups. BA participants had a significantly higher hip circumference contributing to a lower waist-to-hip ratio than WA participants. A high waist-to-hip ratio is associated with visceral adiposity whereas a

low waist-to-hip ratio is associated with greater subcutaneous fat [21]. Larger proportions of visceral fat lend to an increased risk for cardiovascular disease. Interestingly, WA participants did not have a significant difference in blood pressure or pulse rate in comparison to BA participants, regardless of their larger initial levels of visceral adiposity. This may be due to both groups having a substantial BMI rendering waist-to-hip ratio as a less reliable marker for cardiovascular dysfunction.

Though within the normal range for both groups, there were markedly lower liver enzymes in BA participants compared to liver enzymes of WA participants. ALT and AST are liver enzymes whose elevations are indicative of liver damage. While obesity is associated with an elevation of liver enzymes, black populations tend to have lower liver enzymes when compared to white populations [22]. Additionally, liver enzymes are generally elevated in men in comparison to women [22,23]. Despite variations in diagnostic tools, blacks have the lowest prevalence of non-alcoholic fatty liver disease (NAFLD), [24] and non-alcoholic steato-hepatitis (NASH) is inversely associated with being African American, though this finding is somewhat limited by non-histologic diagnosis [25]. Further, the temporal severity of advanced fibrosis is elevated in non-Hispanic whites, whereas in non-Hispanic blacks, the trajectory of severity is reduced [26].

Further, a variant of PNPLA3, a pro-steatotic gene that carries with it a higher incidence of NAFLD occurs with the greatest frequency in Hispanics, followed by non-Hispanic whites, and least in African Americans, may explain the lower prevalence of NAFLD in African Americans despite the prevalence of obesity and diabetes in this population [27]. Despite having lower intrahepatic triglyceride accumulation, once

NAFLD develops, NASH occurs as frequently and as severe as in Caucasian patients [28].

BA participants enrolled in the present study had a lower hematocrit percentage than WA participants, corresponding in pattern to literature showing hematocrit lower in African-Americans than in whites [29]. Though potentially caused by a variety of factors, anemia is a common contributor to low hematocrit and has a higher prevalence in the black population [30]. Platelet counts were also higher in the BA group compared to WA. This is similar to an earlier study in which black women had significantly higher platelet counts than white women [31], particularly applicable to our study as there were no male BA participants.

As expected, participants had noticeable weight loss in comparison to baseline weight with appreciable improvements in BMI, waist circumference, and hip circumference following SG. A recent JAMA Surgery report suggests that BA do not realize the same positive benefits to excess loss of weight as WA [10]. Undoubtedly, given the small sample size of the current study, this facet regarding race was not captured in the data set, though %EWL was greater in the WA participants. Bariatric surgeries are also purported to reduce blood pressure and provide resolution of hypertension along with improvements in heart rate [32,33], as seen in our cohort. It is unknown whether this improvement to cardiovascular health is the result of weight-dependent changes or if the improvement comes from other neural, hormonal, or chemical changes that are weight-independent.

In the current study, IgA and IgM levels were highly variable among participants, both pre-operatively and after six weeks. There was no change to IgA or IgM as a

function of surgery in this short time frame. However, the presence of diabetes pre-operatively was linked with lower IgA levels. Poor glycemic control appears associated with an increase in IgA serum antibodies [34]. Within our data set, the patients identified as diabetic may have more controlled glucose, associated with a reduced IgA in comparison to those individuals who had not been identified to be diabetic. With respect to IgG, BA individuals had higher concentrations at both time points compared to WA, but overall, IgG was dramatically reduced as a result of surgery. IgG is the most common circulating immunoglobulin in the humoral immune system. It binds to pathogens and protects the body from infection by developing a memory of exposure to specific invaders. Black subjects have previously reported to have higher IgG levels in a variety of studies, regardless of context [35,36]. The reason for the higher IgG levels in black subjects remains unknown. The reduction of IgG levels following surgical weight loss is not currently understood.

IgG-specific antibody mediated reactions are a body’s natural defensive reaction to infiltrating food antigens [37]. Following an elimination diet (targeting foods which specifically increased IgG levels), overweight or obese adults were able to decrease IgG antibody titers [38]. Thus, the possibility exists that either as a result of diet choice or genetic factors associated with gastrointestinal permeability, BA have greater levels of IgG that are ameliorated with SG.

CRP is an acute phase, hepatically-derived immune marker for generalized inflammation. As with other markers of inflammation, obesity is associated with higher levels of CRP [39]. Bariatric surgery reduces CRP levels at three months following gastric bypass and as far as twelve months in SG patients [13,40]. Participants in the

current study showed a reduction in CRP levels at six weeks in both BA and WA, although BA individuals had overall higher CRP levels. Interestingly, the change in the reduction of CRP levels at six weeks was comparable in BA and WA when the differential CRP starting levels were controlled for. Overall, this aligns with literature suggesting that black subjects have higher CRP levels when compared to white subjects, and further, that CRP levels are higher in women than in men [41].

TGFβ is an immune cell-derived chemokine which regulates a variety of growth, differentiation, and adhesion cellular processes, in particular the chemotaxis of immune cells. TGFB levels are correlated with obesity in humans [42], and reports show that RYGB decreases TGFβ at one year following surgical weight loss [15]. We show very early reductions in TGFβ in the current work with no differences by race. Circulating levels of TGFβ reductions are not as great as age increased, with older recipients of SG have higher levels of TGFβ. The role of both age and potentially sex in TGFβ production remains to be further studied.

Strengths and Limitations.

The study presented here encompassed patients that were consecutively enrolled in the study. The data collected are representative of short-term weight loss and no information is available about the changes to the immune markers at times greater than six weeks. The trajectories of change in excess weight loss and immune markers may change over time for the population’s samples. One of the significant failures of the study is the consent of BA males. Not only do men seek bariatric surgery at reduced rates compared to women, the historical context of BA male utilization in medical studies functions as a barrier to enrollment for this study.

Overall, the sample size is small and given the current trajectories, some parameters may have benefited from increasing the power of the study. While we focused on a limited number of markers, there are vast numbers of others that may have also been interesting to test and are important for future studies.

Conclusions.

In a subset of patients receiving SG representing the demographics of our region of the country, there were no differences in the magnitude of weight loss following SG at six weeks. BA had greater starting levels of cardiovascular dysfunction, but lower levels of liver dysfunction and reduced tendency for obesity-related lipid disorders. CRP, TGFβ, and IgG were all reduced as a result of SG. IgG was initially elevated in BA in comparison to WA. However, IgG was substantively reduced in BA than WA in the early phase of weight loss. The higher levels of both IgG and CRP in obese BA have significance, given the greater morbidity and mortality of BA individuals in the current viral pandemic [43] where elevations in inflammation appear to exacerbate the severity of symptoms in the BA population [44]. These data suggest that in the early surgical weight loss time frame, markers of immune function are positively improved with SG for both BA and WA but may be improved more so for BA given their higher starting point. Further work is necessary to understand this relationship more adequately.

Acknowledgements

B.E.G. is supported by awards from the Office of the Assistant Secretary of Defense for Health Affairs supported by Award No. W81XWH-16-1-0349 and W81XWH-16-1-0387.

Opinions, interpretations, conclusions, and recommendations are those of the author and are not necessarily endorsed by the Department of Defense.

The Mississippi Center of Excellence in Perinatal Research (MS-CEPR)-COBRE P20GM121334, also supported B.E.G and S.T.L. Research reported in this publication was also supported by the National Institute of General Medical Sciences of the National Institutes of Health under Award Number 1U54GM115428. The content is solely the responsibility of the authors and does not necessarily represent the official views of the National Institutes of Health.

Author Contributions

Conceptualization: CLP, SG, and BEG
Data Curation: CLP and BEG
Formal Analysis: CLP, TTL, SL, and BEG
Funding Acquisition: BEG
Investigation: CLP, KDV, and BEG
Methodology: CLP and BEG
Project Administration: KDV and BEG
Resources: KDV and BEG
Supervision: BEG
Validation: CLP and BEG
Visualization: CLP, TTL, SL, and BEG
Writing/Original Draft: CLP, TTL, SL, and BEG
Writing/Review and Editing: CLP, BAW, TTL, SL, SG, KDV, and BEG

Conflicts of interest

CLP, BAW, AD, TTL, STL, SCG, KDV, and BEG have no financial, personal, or professional conflicts of interest.

REFERENCES

1. Zhang, Q.; Wang, Y.; Huang, E.S. Changes in racial/ethnic disparities in the prevalence of Type 2 diabetes by obesity level among US adults. *Ethnicity & health* **2009**, *14*, 439-457, doi:10.1080/13557850802699155.
2. Funk, L.M.; Shan, Y.; Voils, C.I.; Kloke, J.; Hanrahan, L.P. Electronic Health Record Data Versus the National Health and Nutrition Examination Survey (NHANES): A Comparison of Overweight and Obesity Rates. *Medical care* **2017**, *55*, 598-605, doi:10.1097/mlr.0000000000000693.
3. Guo, F.; He, D.; Zhang, W.; Walton, R.G. Trends in prevalence, awareness, management, and control of hypertension among United States adults, 1999 to 2010. *Journal of the American College of Cardiology* **2012**, *60*, 599-606, doi:10.1016/j.jacc.2012.04.026.
4. Chow, E.; Foster, H.; Gonzalez, V.; McIver, L. The Disparate Impact of Diabetes on Racial/Ethnic Minority Populations. *Clinical Diabetes* **2012**, *30*, 130-133, doi:10.2337/diaclin.30.3.130.
5. Schmeer, K.K.; Tarrence, J. Racial-ethnic Disparities in Inflammation: Evidence of Weathering in Childhood? *J Health Soc Behav* **2018**, *59*, 411-428, doi:10.1177/0022146518784592.
6. Paalani, M.; Lee, J.W.; Haddad, E.; Tonstad, S. Determinants of inflammatory markers in a bi-ethnic population. *Ethnicity & disease* **2011**, *21*, 142-149.
7. Park, N.-J.; Kang, D.-H. Inflammatory Cytokine Levels and Breast Cancer Risk Factors: Racial Differences of Healthy Caucasian and African American Women. *Oncology nursing forum* **2013**, *40*, A1-A11, doi:10.1188/13.ONF.40-05AP.

8. Irwin, M. Effects of sleep and sleep loss on immunity and cytokines. *Brain, behavior, and immunity* **2002**, *16*, 503-512, doi:10.1016/s0889-1591(02)00003-x.

9. Mulla, C.M.; Middelbeek, R.J.W.; Patti, M.-E. Mechanisms of weight loss and improved metabolism following bariatric surgery. *Ann N Y Acad Sci* **2018**, *1411*, 53-64, doi:10.1111/nyas.13409.

10. Wood, M.H.; Carlin, A.M.; Ghaferi, A.A.; Varban, O.A.; Hawasli, A.; Bonham, A.J.; Birkmeyer, N.J.; Finks, J.F. Association of Race With Bariatric Surgery Outcomes. *JAMA surgery* **2019**, 10.1001/jamasurg.2019.0029, e190029, doi:10.1001/jamasurg.2019.0029.

11. Zhang, C.; Zhang, J.; Liu, Z.; Zhou, Z. More than an Anti-diabetic Bariatric Surgery, Metabolic Surgery Alleviates Systemic and Local Inflammation in Obesity. *Obes Surg* **2018**, *28*, 3658-3668, doi:10.1007/s11695-018-3400-z.

12. Askarpour, M.; Khani, D.; Sheikhi, A.; Ghaedi, E.; Alizadeh, S. Effect of Bariatric Surgery on Serum Inflammatory Factors of Obese Patients: a Systematic Review and Meta-Analysis. *Obes Surg* **2019**, *29*, 2631-2647, doi:10.1007/s11695-019-03926-0.

13. Zhang, C.; Zhang, J.; Liu, W.; Chen, X.; Liu, Z.; Zhou, Z. Improvements in humoral immune function and glucolipid metabolism after laparoscopic sleeve gastrectomy in patients with obesity. *Surgery for Obesity and Related Diseases* **2019**, *15*, 1455-1463, doi:https://doi.org/10.1016/j.soard.2019.05.021.

14. Hakeam, H.A.; O'Regan, P.J.; Salem, A.M.; Bamehriz, F.Y.; Jomaa, L.F. Inhibition of C-reactive protein in morbidly obese patients after laparoscopic sleeve gastrectomy. *Obesity surgery* **2009**, *19*, 456-460.

15. Lindegaard, K.K.; Jorgensen, N.B.; Just, R.; Heegaard, P.M.; Madsbad, S. Effects of Roux-en-Y gastric bypass on fasting and postprandial inflammation-related parameters in obese subjects with normal glucose tolerance and in obese subjects with type 2 diabetes. *Diabetology & metabolic syndrome* **2015**, *7*, 12, doi:10.1186/s13098-015-0012-9.

16. Fathy, S.M.; Morshed, G. Peripheral blood lymphocyte subsets (CD4+, CD8+ T cells), leptin level and weight loss after laparoscopic greater curvature plication in morbidly obese patients. *Archives of medical science : AMS* **2014**, *10*, 886-890, doi:10.5114/aoms.2014.46209.

17. Aron-Wisnewsky, J.; Tordjman, J.; Poitou, C.; Darakhshan, F.; Hugol, D.; Basdevant, A.; Aissat, A.; Guerre-Millo, M.I.; Clément, K. Human Adipose Tissue Macrophages: M1 and M2 Cell Surface Markers in Subcutaneous and Omental Depots and after Weight Loss. *The Journal of Clinical Endocrinology & Metabolism* **2009**, *94*, 4619-4623, doi:10.1210/jc.2009-0925.

18. Poitou, C.; Dalmas, E.; Renovato, M.; Benhamo, V.; Hajduch, F.; Abdennour, M.; Kahn, J.-F.; Veyrie, N.; Rizkalla, S.; Fridman, W.-H., et al. CD14dimCD16+ and CD14+CD16+ Monocytes in Obesity and During Weight Loss. *Arteriosclerosis, thrombosis, and vascular biology* **2011**, *31*, 2322-2330, doi:10.1161/ATVBAHA.111.230979.

19. Harris, P.A.; Taylor, R.; Thielke, R.; Payne, J.; Gonzalez, N.; Conde, J.G. Research electronic data capture (REDCap)--a metadata-driven methodology and workflow process for providing translational research informatics support.

Journal of biomedical informatics **2009**, *42*, 377-381, doi:10.1016/j.jbi.2008.08.010.

20. Phillips, C.L.; Grayson, B.E. The immune remodel: Weight loss-mediated inflammatory changes to obesity. *Experimental biology and medicine (Maywood, N.J.)* **2020**, *245*, 109-121, doi:10.1177/1535370219900185.
21. Ashwell, M.; Cole, T.J.; Dixon, A.K. Obesity: new insight into the anthropometric classification of fat distribution shown by computed tomography. *Br Med J (Clin Res Ed)* **1985**, *290*, 1692-1694, doi:10.1136/bmj.290.6483.1692.
22. Schneider, A.L.C.; Lazo, M.; Ndumele, C.E.; Pankow, J.S.; Coresh, J.; Clark, J.M.; Selvin, E. Liver enzymes, race, gender and diabetes risk: the Atherosclerosis Risk in Communities (ARIC) Study. *Diabet Med* **2013**, *30*, 926-933, doi:10.1111/dme.12187.
23. Clark, J.M.; Brancati, F.L.; Diehl, A.M. The prevalence and etiology of elevated aminotransferase levels in the United States. *Am J Gastroenterol* **2003**, *98*, 960-967, doi:10.1111/j.1572-0241.2003.07486.x.
24. Pan, J.-J.; Fallon, M.B. Gender and racial differences in nonalcoholic fatty liver disease. *World journal of hepatology* **2014**, *6*, 274-283, doi:10.4254/wjh.v6.i5.274.
25. Younossi, Z.M.; Stepanova, M.; Negro, F.; Hallaji, S.; Younossi, Y.; Lam, B.; Srishord, M. Nonalcoholic fatty liver disease in lean individuals in the United States. *Medicine* **2012**, *91*, 319-327, doi:10.1097/MD.0b013e3182779d49.
26. Kim, D.; Kim, W.; Adejumo, A.C.; Cholankeril, G.; Tighe, S.P.; Wong, R.J.; Gonzalez, S.A.; Harrison, S.A.; Younossi, Z.M.; Ahmed, A. Race/ethnicity-based

temporal changes in prevalence of NAFLD-related advanced fibrosis in the United States, 2005-2016. *Hepatol Int* **2019**, *13*, 205-213, doi:10.1007/s12072-018-09926-z.

27. Iqbal, U.; Perumpail, B.J.; Akhtar, D.; Kim, D.; Ahmed, A. The Epidemiology, Risk Profiling and Diagnostic Challenges of Nonalcoholic Fatty Liver Disease. *Medicines (Basel)* **2019**, *6*, 41, doi:10.3390/medicines6010041.
28. Bril, F.; Portillo-Sanchez, P.; Liu, I.C.; Kalavalapalli, S.; Dayton, K.; Cusi, K. Clinical and Histologic Characterization of Nonalcoholic Steatohepatitis in African American Patients. *Diabetes Care* **2018**, *41*, 187-192, doi:10.2337/dc17-1349.
29. Beutler, E.; West, C. Hematologic differences between African-Americans and whites: the roles of iron deficiency and alpha-thalassemia on hemoglobin levels and mean corpuscular volume. *Blood* **2005**, *106*, 740-745, doi:10.1182/blood-2005-02-0713.
30. Le, C.H. The Prevalence of Anemia and Moderate-Severe Anemia in the US Population (NHANES 2003-2012). *PloS one* **2016**, *11*, e0166635, doi:10.1371/journal.pone.0166635.
31. Saxena, S.; Cramer, A.D.; Weiner, J.M.; Carmel, R. Platelet counts in three racial groups. *Am J Clin Pathol* **1987**, *88*, 106-109, doi:10.1093/ajcp/88.1.106.
32. Wilhelm, S.M.; Young, J.; Kale-Pradhan, P.B. Effect of bariatric surgery on hypertension: a meta-analysis. *Ann Pharmacother* **2014**, *48*, 674-682, doi:10.1177/1060028014529260.
33. Nault, I.; Nadreau, E.; Paquet, C.; Brassard, P.; Marceau, P.; Marceau, S.; Biron, S.; Hould, F.; Lebel, S.; Richard, D., et al. Impact of bariatric surgery--induced

weight loss on heart rate variability. *Metabolism: clinical and experimental* **2007**, *56*, 1425-1430, doi:10.1016/j.metabol.2007.06.006.

34. Awartani, F. Serum immunoglobulin levels in type 2 diabetes patients with chronic periodontitis. *J Contemp Dent Pract* **2010**, *11*, 001-008.

35. Lucey, D.R.; Hendrix, C.W.; Andrzejewski, C.; Melcher, G.P.; Butzin, C.A.; Henry, R.; Wians, F.H., Jr.; Boswell, R.N. Comparison by race of total serum IgG, IgA, and IgM with CD4+ T-cell counts in North American persons infected with the human immunodeficiency virus type 1. *Journal of acquired immune deficiency syndromes* **1992**, *5*, 325-332.

36. Tollerud, D.J.; Brown, L.M.; Blattner, W.A.; Weiss, S.T.; Maloney, E.M.; Kurman, C.C.; Nelson, D.L.; Hoover, R.N. Racial differences in serum immunoglobulin levels: relationship to cigarette smoking, T-cell subsets, and soluble interleukin-2 receptors. *Journal of clinical laboratory analysis* **1995**, *9*, 37-41, doi:10.1002/jcla.1860090107.

37. Gocki, J.; Bartuzi, Z. Role of immunoglobulin G antibodies in diagnosis of food allergy. *Postepy Dermatol Alergol* **2016**, *33*, 253-256, doi:10.5114/ada.2016.61600.

38. Neuendorf, R.; Corn, J.; Hanes, D.; Bradley, R. Impact of Food Immunoglobulin G-Based Elimination Diet on Subsequent Food Immunoglobulin G and Quality of Life in Overweight/Obese Adults. *J Altern Complement Med* **2019**, *25*, 241-248, doi:10.1089/acm.2018.0310.

39. Paepegaey, A.C.; Genser, L.; Bouillot, J.L.; Oppert, J.M.; Clement, K.; Poitou, C. High levels of CRP in morbid obesity: the central role of adipose tissue and

lessons for clinical practice before and after bariatric surgery. *Surg Obes Relat Dis* **2015**, *11*, 148-154, doi:10.1016/j.soard.2014.06.010.

40. Zagorski, S.M.; Papa, N.N.; Chung, M.H. The effect of weight loss after gastric bypass on C-reactive protein levels. *Surg Obes Relat Dis* **2005**, *1*, 81-85, doi:10.1016/j.soard.2005.01.001.

41. Khera, A.; McGuire, D.K.; Murphy, S.A.; Stanek, H.G.; Das, S.R.; Vongpatanasin, W.; Wians, F.H.; Grundy, S.M.; de Lemos, J.A. Race and Gender Differences in C-Reactive Protein Levels. *Journal of the American College of Cardiology* **2005**, *46*, 464-469, doi:https://doi.org/10.1016/j.jacc.2005.04.051.

42. Yadav, H.; Quijano, C.; Kamaraju, A.K.; Gavrilova, O.; Malek, R.; Chen, W.; Zerfas, P.; Zhigang, D.; Wright, E.C.; Stuelten, C., et al. Protection from obesity and diabetes by blockade of TGF-beta/Smad3 signaling. *Cell Metab* **2011**, *14*, 67-79, doi:10.1016/j.cmet.2011.04.013.

43. Price-Haywood, E.G.; Burton, J.; Fort, D.; Seoane, L. Hospitalization and Mortality among Black Patients and White Patients with Covid-19. *The New England journal of medicine* **2020**, *382*, 2534-2543, doi:10.1056/NEJMsa2011686.

44. García, L.F. Immune Response, Inflammation, and the Clinical Spectrum of COVID-19. *Front Immunol* **2020**, *11*, 1441, doi:10.3389/fimmu.2020.01441.

FIGURE LEGENDS

Table 1: Baseline comorbidities of study participants. Data presented as percentages. Comparisons made using Chi-squared test.

Table 2: Pre-operative and post-operative characteristics of participants. Data presented as mean ± SEM. Pre-operative values between BA and WA compared using student T test (Column A vs. B). Two-way Anova was used to compare variables of race and time. Boldface designates statistical significance 0.05. BP (blood pressure); ALT (alanine transaminase); AST (aspartate aminotransferase); WBC (white blood cells).

Table 3: Modelling results of pre-operative immune markers. Displayed are beta coefficients, p-values, and 95% confidence intervals. Coefficients for age, BMI, SBP, and DBP correspond to one-unit increases. BMI (body mass index); SBP (systolic blood pressure); DBP (diastolic blood pressure); CRP (c-reactive protein); IgA (immunoglobulin A); IgG (immunoglobulin G); IgM (immunoglobulin M); TGFB (transforming growth factor beta).

Table 4: Modelling results for weight change. Displayed are beta coefficients, p-values, and 95% confidence intervals. Change was defined as post-op minus pre-op, ergo a positive coefficient indicates, on average, less weight loss. Coefficient for age corresponds to one year increase. DM (diabetes mellitus).

Figure 1: Immunoglobulins and immune markers pre- and post-SG. (**A**) Plasma IgA (**B**) Plasma IgG (**C**) IgM (**D**) CRP (**E**) TGFβ. Data presented as mean ± SEM. Two-way Anova was used to compare variables of race and time. IgA (immunoglobulin A); IgG (immunoglobulin G); IgM (immunoglobulin M); CRP (c-reactive protein); TGFB (transforming growth factor beta).

Table 5: Modelling results for immune marker change. Displayed are beta coefficients, p-values, and 95% confidence intervals. Change was defined as post-op minus pre-op, ergo a positive coefficient indicates, on average, less reduction. Coefficients for age, BMI, SBP, and DBP correspond to one-unit increases. HTN (hypertension); DM (diabetes mellitus); BMI (body mass index); SBP (systolic blood pressure); DBP (diastolic blood pressure); CRP (c-reactive protein); IgA (immunoglobulin A); IgG (immunoglobulin G); IgM (immunoglobulin M); TGFB (transforming growth factor beta).

Click here to access/download;Figure;Phillips POM Figures.pptx

TABLE 1

Participant Characteristics	BA	%	WA	%	Statistics
Hypertension (Y/Total)	22/58	*91.6%*	15/58	*44.1%*	*chi^2=13.77;* ***p<0.001***
Hyperlipidemia (Y/Total)	4/58	*16.7%*	13/58	*38.2%*	*chi^2=3.16; p=0.07*
Diabetes (Y/Total)	9/58	*37.5%*	7/58	*20.5%*	*chi^2=2.01; p=0.16*
Obstructive Sleep Apnea (Y/Total)	11/58	*45.8%*	13/58	*38.2%*	*chi^2=0.33; p=0.56*

TABLE 2

	BA Pre-op (A)			WA Pre-op (B)			BA 6 wks post-op (C)			WA 6 wks post-op (D)			STATISTIC Student's T	STATISTIC Two-way ANOVA
	**Mean**	**±**	**SEM**	**Mean**	**±**	**SEM**	**Mean**	**±**	**SEM**	**Mean**	**±**	**SEM**	A vs B	time =AB vs CD; race= AC vs. BD
	**n=24**			**n=24**			**n=24**			**n=24**				
Body weight (kg)	127.6	±	4.09	123.8	±	4.02	113.4	±	3.73	108.4	±	3.52	p=0.5206	***p(time)<0.0001***; p(race)=0.4292
Body Mass Index (BMI)	47.5	±	1.27	44.7	±	1.03	42.9	±	1.50	39.5	±	1.09	p=0.0934	***p(time)<0.0001***; p(race)=0.0659
Waist Circumference (cm)	124.5	±	2.87	128.0	±	2.73	115.2	±	2.83	123.7	±	2.52	p=0.4047	***p(time)<0.0001***; p(race)<0.1826
Hip Circumference (cm)	134.8	±	2.78	128.2	±	1.99	112.9	±	2.32	122.5	±	1.87	***p<0.05***	***p(time)<0.0001***; p(race)<0.8606
Systolic BP (mm Hg)	143.3	±	4.31	138.6	±	1.91	134.1	±	2.65	127.7	±	3.03	p=0.2744	***p(time)<0.0001***; p(race)<0.1230
Diastolic BP (mm Hg)	85.4	±	1.97	81.6	±	2.06	84.8	±	1.55	77.4	±	2.69	p=0.2051	p(time)=0.1850; ***p(race)<0.05***
Pulse (bpm)	83.6	±	3.42	79.1	±	2.05	80.0	±	2.81	74.4	±	1.99	p=0.2386	***p(time)<0.05***; p(race)=0.0661
Temperature (◦ C)	97.7	±	0.17	97.5	±	0.14	97.6	±	0.13	96.8	±	0.94	p=0.3567	p(time)=0.5813; p(race)= 0.278
ALT (IU/L)	18.0	±	1.69	36.5	±	3.82	N/A			N/A			***p=0.0005***	N/A
AST (IU/L)	17.7	±	1.15	29.6	±	3.05	N/A			N/A			***p=0.0033***	N/A
Billirubin (mg/dL)	0.4	±	0.06	0.5	±	0.04	N/A			N/A			p=0.3286	N/A
Creatinine (mg/dL)	0.8	±	0.03	0.8	±	0.02	N/A			N/A			p=0.3576	N/A
Glucose (mg/dL)	104.9	±	5.11	113.6	±	5.88	N/A			N/A			p=0.2996	N/A
WBC (x10 /ul)	8.0	±	0.54	7.3	±	0.30	N/A			N/A			p=0.2053	N/A
Hematocrit %	38.7	±	0.77	41.9	±	0.63	N/A			N/A			***p=0.0026***	N/A
Platelets (x10 /ul)	312.5	±	16.28	266.0	±	12.13	N/A			N/A			***p=0.0234***	N/A

TABLE 3

Models for PreOp Immune Markers

	CRP	IgA	IgG	IgM	TGFB
**Age**	0.01 p=0.741 (-0.03, 0.05)	0.25 p=0.442 (-0.39, 0.89)	27.25 p=0.202 (-14.65, 69.14)	-2.03 p=0.219 (-5.25, 1.20)	-0.10 p=0.333 (-0.31, 0.11)
**Black**	-0.1 p=0.829 (-1.01, 0.81)	3.70 p=0.566 (-8.95, 16.37)	1377.36 p=0.001 (569.83, 2184.89)	-22.78 p=0.625 (-114.11, 68.55)	-0.72 p=0.729 (-4.82, 3.37)
**Hypertension**	-0.06 p=0.879 (-0.85, 0.73)	3.45 p=0.637 (-10.86, 17.76)	-326.06 p=0.440 (-1154.00, 501.88)	28.6 p=0.537 (-62.30, 119.49)	2.10 p=0.480 (-3.71, 7.91)
**Hyperlipidemia**	-1.20 p=0.001 (-1.88, -0.51)	8.14 p=0.279 (-6.60, 22.88)	634.20 p=0.133 (-193.05, 1461.43)	-20.84 p=0.614 (-101.77, 60.09)	3.71 p=0.220 (-2.22, 9.65)
**Sleep Apnea**	0.03 p=0.945 (-0.79, 0.85)	7.90 p=0.266 (-6.04, 21.85)	-228.24 p=0.597 (-1074.56, 618.08)	-14.01 p= 0.634 (-71.72, 43.71)	-0.89 p=0.721 (-5.78, 4.00)
**Diabetes**	0.46 p=0.170 (-0.20, 1.13)	-17.33 p=0.029 (-32.88, -1.79)	-610.98 p=0.152 (-1446.08, 224.12)	-18.68 p=0.664 (-102.91, 65.55)	1.29 p=0.670 (-4.64, 7.22)
**PreOp BMI**	0.06 p=0.224 (-0.03, 0.14)	-0.01 p=0.978 (-1.03, 1.00)	59.37 p=0.110 (-13.36, 132.09)	-0.49 p=0.901 (-8.28, 7.29)	-0.08 p=0.710 (-0.49, 0.34)
**PreOp SBP**	0.00 p=0.731 (-0.02, 0.03)	-0.29 p=0.230 (-0.78, 0.19)	1.73 p=0.916 (-30.32, 33.78)	-0.87 p=0.534 (-3.62, 1.88)	0.07 p=0.447 (-0.10, 0.23)
**PreOp DBP**	-0.04 p=0.032 (-0.08, 0.00)	0.03 p=0.933 (-.65, .71)	15.22 p=0.422 (-21.94, 52.39)	1.35 p=0.453 (-2.17, 4.87)	-0.06 p=0.586 (-0.29, 0.17)

TABLE 4

Models for Weight Change

	Weight Change (kg)	BMI Change
Age	0.10 ***p=0.045*** (0.00, 0.20)	0.04 ***p=0.047*** (0.00, 0.07)
Black	-0.09 p=0.935 (-2.24, 2.07)	-0.15 p=0.703 (-0.90, 0.61)
Hypertension	0.67 p=0.543 (-1.54, 2.88)	0.53 p=0.180 (-0.25, 1.31)
Hyperlipidemia	0.75 p=0.479 (-1.37, 2.88)	-0.20 p=0.585 (-0.95, 0.54)
Sleep Apnea	-1.28 p=0.223 (-3.36, .80)	-0.19 p=0.599 (-0.93, 0.54)
DM	0.18 p=0.869 (-2.03, 2.40)	0.05 p=0.899 (-0.73, 0.83)

FIGURE 1

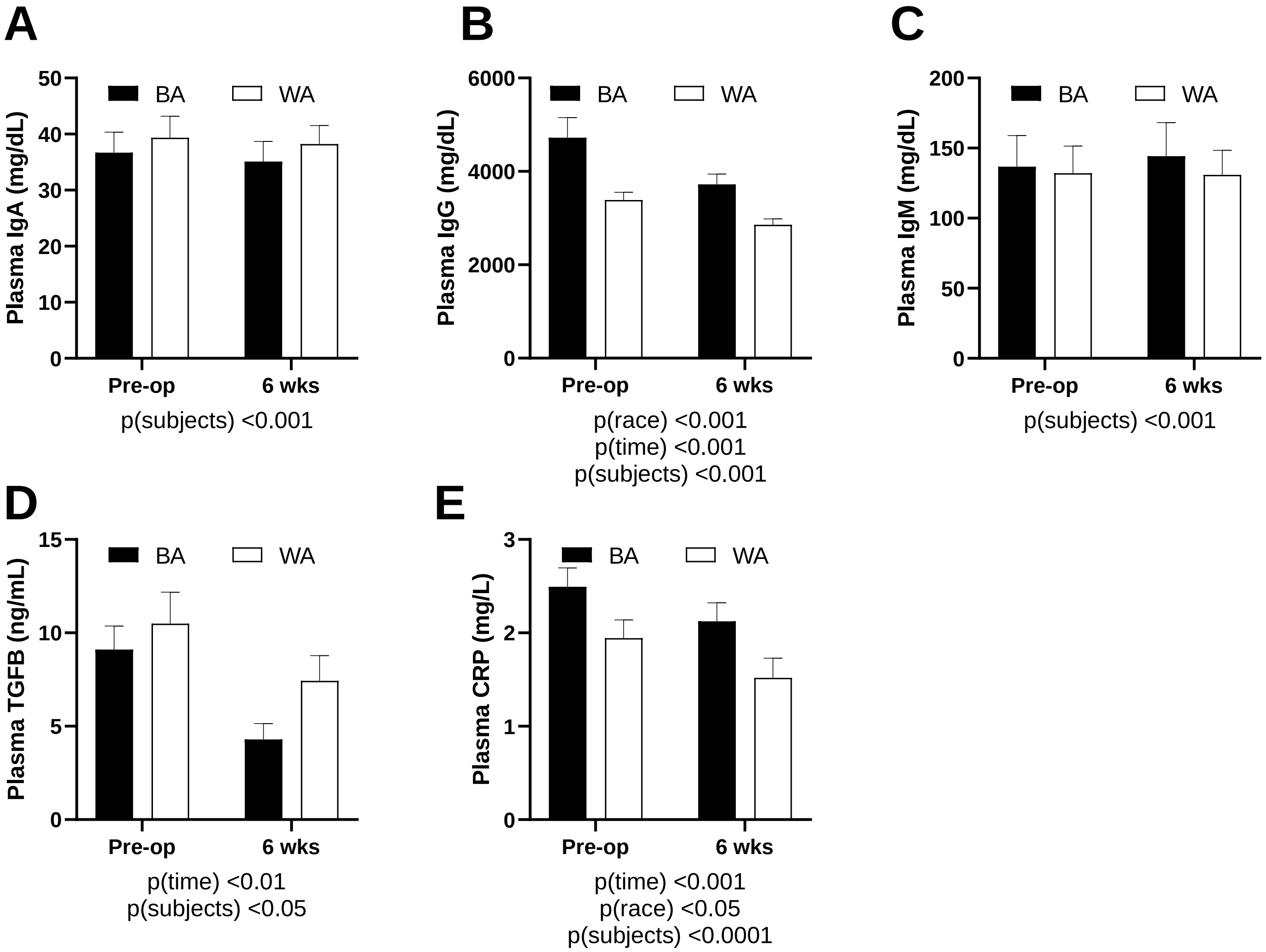
A
Plasma IgA (mg/dL)
BA
WA
Pre-op
6 wks
p(subjects) <0.001
B
Plasma IgG (mg/dL)
BA
WA
Pre-op
6 wks
p(race) <0.001
p(time) <0.001
p(subjects) <0.001
C
Plasma IgM (mg/dL)
BA
WA
Pre-op
6 wks
p(subjects) <0.001
D
Plasma TGFB (ng/mL)
BA
WA
Pre-op
6 wks
p(time) <0.01
p(subjects) <0.05
E
Plasma CRP (mg/L)
BA
WA
Pre-op
6 wks
p(time) <0.001
p(race) <0.05
p(subjects) <0.0001

TABLE 5

Models for Immune Marker Change

	Δ CRP	Δ IgA	Δ IgG	Δ IgM	ΔTGFB
**Age**	-0.01 p=0.292 (-0.04, 0.01)	0.12 p=0.636 (-0.37, 0.60)	6.36 p=0.767 (-36.60, 49.32)	0.01 p=0.980 (-1.07, 1.09)	0.35 p=0.030 (0.04, 0.66)
**Black**	0.22 p=0.366 (-0.27, 0.71)	-0.14 p=0.977 (-9.87, 9.59)	-1108.04 p=0.012 (-1963.49, -252.60)	3.13 p=0.771 (-18.38, 24.65)	0.91 p=0.771 (-5.34, 7.15)
**HTN**	-0.14 p=0.579 (-0.65, 0.36)	-2.39 p=0.633 (-12.42, 7.63)	606.87 p=0.173 (-274.93, 1488.67)	-2.48 p=0.823 (-24.66, 19.70)	-6.28 p=0.055 (-12.71, 0.15)
**Hyperlipidemia**	0.22 p=0.374 (-0.27, 0.71)	-4.76 p=0.329 (-14.47, 4.95)	-608.68 p=0.158 (-1462.35, 244.99)	-17.47 p=0.108 (-38.94, 4.00)	-3.75 p=0.232 (-9.98, 2.48)
**Sleep Apnea**	-0.25 p=0.291 (-0.72, 0.22)	0.62 p=0.895 (-8.77, 10.01)	-235.55 p=0.569 (-1061.37, 590.28)	16.51 p=0.116 (-4.25, 37.29)	-0.08 p=0.978 (-6.11, 5.94)
**DM**	0.15 p=0.560 (-0.36, 0.65)	0.09 p=0.986 (-9.89, 10.07)	745.39 p=0.094 (-132.33, 1623.10)	23.81 p=0.035 (1.74, 45.88)	1.09 p=0.734 (-5.32, 7.49)
**BMI Δ**	-0.12 p=0.223 (-0.31, 0.07)	0.05 p=0.979 (-3.73, 3.83)	301.21 p=0.074 (-30.90, 633.32)	-5.39 p=0.201 (-13.74, 2.97)	-0.75 p=0.537 (-3.17, 1.67)
**SBP Δ**	-0.01 p=0.145 (-0.02, 0.00)	0.07 p=0.633 (-0.21, 0.35)	11.41 p=0.355 (-13.18, 36.00)	-0.13 p=0.684 (-0.74, 0.49)	0.11 p=0.233 (-0.07, 0.29)
**DBP Δ**	0.01 p=0.322 (-0.01, 0.03)	0.02 p=0.915 (-0.33, 0.37)	5.88 p=0.705 (-25.14, 36.89)	-0.22 p=0.569 (-1.00, 0.56)	-0.07 p=0.542 (-0.30, 0.16)
