# Supplementary material for: Immune marker reductions in black and white Americans following sleeve gastrectomy in the short-term phase of surgical weight loss
Source: PLoS One. 2023 Jul 25;18(7):e0288868. doi: 10.1371/journal.pone.0288868 (PMC10368278; doi:10.1371/journal.pone.0288868)
Supplement: Attachment — Submitted filename: Comments (2).docx [file pone.0288868.s002.docx]

PONE-D-22-23672

The authors of the paper determined if IgG, IgM, IgA, TGFβ, and CRP change similarly in black and white Americans after sleeve gastrectomy.

While the rationale of the study seems justified, there are some important problems in the article. Aim, hypothesis, study questions, statistical methods to test these questions, and a logical presentation of the results and discussion unfortunately all seem weak.

What was the reason for selecting IgG, IgM, IgA, TGFβ, and CRP, specifically?

Introduction: First paragraph. References are missing.

The statistical methods are unclear, and it is difficult to follow what is the study question for which each of the tests is performed.

For example:

‘In addition to the adjusters listed below’

Please clarify what are these adjusters? Below is mentioned the adjusters for change, as well as for ‘all models were adjusted for…’

‘Weight change was modeled using ordinary least squares on the difference in weight post-op vs. pre-op. Similar models were used to model immune marker change, additionally adjusting for BMI change, systolic change, and diastolic change.’

It is unclear where these models were used (what is the study question, predictor, outcome).

The models are not adjusted for multiple testing. For example, in Table 5, none of the p values would likely remain significant if multiple comparisons were taken into account.

The results section is inconsistent.

There seems to be no real logic and in every paragraph, the topics jump from one area to another.

For example, the first paragraph of the results mixes sex, age and BA/Wa comparison.

Row 149-

The percentages are unclear regarding total, f/m.

BA had no males but 6/34 of WA were males. Was there a difference in immune markers or clinical conditions between the sexes in WA?

The next parag (row 157) continues with ‘Pre-operative Characteristics of BA and WA Participants’, but wasn’t there already preop comparisons in the previous parag?

The inconsistency continues in the next parag. The text jumps from influence of clinical characteristics to comparisons of BA/WA and back to clinical characteristics.

In the text, what test is performed for ‘influence’ or ‘association’. The text also refers to ‘correlation’. What is that test?

The immune marker results are drown to the quite expected weight + clinical characteristic text.

‘Plasma IgG varied significantly by race with BA having higher levels of IgG than WA, p<0.001.’ What time point is this referring to?

‘Furthermore, BA had greater reductions in IgG, with an average reduction of 1108 mg/dL as a result of SG in comparison to WA (TABLE 5).’

Is the %change in IgG between BA/WA different. Usually, reductions are greater if the baseline level is higher.

Please use exact p values instead of p<0.05 etc.

Discussion

The discussion lacks focus. For example, there are several paragraphs of ALT, NAFLD, hematocrit, platelet count, although these were not mentioned in the results text and are not relevant for the study question.

The discussion also presents new data that is not in the results, like:

‘Participants in the current study showed a reduction in CRP levels at six weeks in both BA and WA, although BA individuals had overall higher CRP levels.’

CRP was not mentioned in the results.

After the discussion, the biological or clinical conclusion of the study remains unclear.

Table 1, what does Y mean?

What do the percentages mean?

In Figure 1: What is p(subjects). What time point is meant for p(race)?

Figure 1 appears between the tables
